# Linking prevention and adherence: a behavioural study on treatment compliance to preventing TB transmission

**DOI:** 10.5588/ijtldopen.26.0117

**Published:** 2026-10-12

**Authors:** A.N. Rakhmani, J. KaewKungwal

**Affiliations:** 1 Department Family Medicine, Faculty of Medicine, Universitas Brawijaya, Malang, Indonesia;; 2 Faculty of Medicine, Universitas Brawijaya, Malang, Indonesia;; 3 Department Tropical Hygiene, Faculty of Tropical Medicine, Mahidol University, Thailand.

**Keywords:** tuberculosis, Indonesia, primary health care

## Abstract

**OBJECTIVES:**

To assess the concordance between TB prevention practices and treatment adherence and identify determinants of a combined prevention and adherence behaviour.

**METHODS:**

A cross-sectional study was conducted among 147 TB patients attending seven urban primary health centres in Malang, Indonesia (January to June 2025). Prevention behaviours and treatment adherence were assessed using structured questionnaire Agreement between prevention practices and adherence was analysed using weighted Cohen kappa and Gwet AC. Determinants of the combined behavioural categories were identified using multinomial logistic regression.

**RESULTS:**

High prevention behaviour and high treatment adherence was observed in 52% and 57%. Agreement between these two behaviours was moderate. Results showed 34% of patients consistently had high prevention and adherence behaviour, 53% showed moderate behaviour, and 13% showed low behaviour. Moderate combined behaviour was associated with gender and TB knowledge. High combined behaviour was significantly associated with higher education, greater income, stronger family functioning, more positive attitudes, and higher TB knowledge.

**CONCLUSION:**

Routine educational programmes are needed for patients and their families during the intensive phase of treatment, along with individualised family assistance through personalised education sessions when patients visit primary health centres to collect their medications.

TB remains a significant public health burden in Indonesia, which ranks among the countries with the highest TB incidence globally.^1^ According to the WHO Global Tuberculosis Report 2025, eight countries contributed approximately 67% of all TB cases worldwide and Indonesia alone contributing approximately 10% of the global burden, ranked second after India.^1^ National data reinforce the severity of Indonesia’s TB incidence, and in 2023 this was estimated at 387/100,000 population.^2^ TB affects all age groups and sexes, but the highest burden is among adult men aged ≥15 years, accounting for 54% of global TB cases in 2024, while adult women accounted for 35% of cases.^1^

Effective TB control depends on two interrelated pillars: preventive practices and treatment adherence. Prevention of TB transmission requires consistent patient behaviours such as mask use, proper respiratory hygiene, and maintaining adequate indoor ventilation. However, the adoption of these practices is influenced by various factors, including gender, knowledge, attitude, supportive behaviours, and adherence to medication.^3–5^ Meanwhile, adherence to the prolonged multidrug regimen remains a formidable challenge. Studies consistently highlight that adherence to treatment is essential for successful therapeutic outcomes, whereas non-adherence not only results in poor clinical consequences but also poses a major obstacle to global TB eradication efforts with increasing drug resistance.^6,7^ Nonetheless, the long- term course of the treatment might expose patients for non-adherence.^8^ National statistics show that overall treatment success for TB in Indonesia reached 86% in 2022, yet outcomes for multidrug-resistant TB patients were significantly lower at 51%.^9^ Complementing this, cross-sectional studies from Thailand and Indonesia show that patients who practice prevention behaviours against TB transmission tend to demonstrate better treatment adherence.^3,4^ Factors influencing TB treatment adherence and transmission-prevention behaviours are multifactorial, spanning individual, familial, social, and structural levels.^3,4,6,7^ Studies have identified a range of determinants, including patients’ understanding of TB and its treatment duration, financial constraints, access to health care, stigma, medication side effects, long treatment courses, family support, and others.

Understanding patient behaviours related to TB transmission prevention and treatment adherence, as well as the factors influencing these behaviours, is therefore essential. The main objectives of this study were: 1) to evaluate the concordance between patients’ TB transmission-prevention behaviours and their treatment adherence and 2) to identify determinants of a composite TB prevention–adherence behaviour among patients in Malang District, East Java Province, Indonesia, an area experiencing rising TB cases.

## METHODS

This study was conducted in seven primary health care centres located in the urban area of Malang, Indonesia, between January and June 2025. A cross-sectional design was employed, involving 147 patients diagnosed with TB who came to primary health care and had already started the treatment.

### Data collection

A structured questionnaire was developed and administered to patients during their visits to TB clinics. Knowledge was measured using items adapted from the WHO instrument,^10^ with a Cronbach’s alpha of 0.91 in this study. Meanwhile, attitude and practice was adapted and modified from previous studies,^5^ yielding a Cronbach’s alpha of 0.85 and 0.66, respectively, in this study. Family function was evaluated using the Family APGAR questionnaire. Scores range from 0 to 10, with family function classified as good (8–10), moderate (4–7), or low (0–3). Treatment adherence was measured using the standard MMAS-8 scale as applied in previous studies.^11^ Adherence scores were classified as high (8 or 100%), moderate (6–7 or 75%–99.9%), or low (0–5, <75%). Using commonly applied cut-off points in public health research,^12^ KAP scores were converted to percentages and classified as good (>80%), moderate (60–79%), or low (<60%).

### Statistical analysis

The sample size was determined using well-established rules of thumb recommended in the literature.^13^ Descriptive statistics were presented using frequencies and percentages. Kappa statistics were used to assess the concordance between patients’ TB transmission-prevention behaviours and treatment adherence. Both weighted Cohen’s kappa (quadratic) and Gwet’s agreement coefficient (AC) were applied. Gwet’s AC was also used because it provides a more stable estimate of agreement when observed agreement is high or when marginal distributions are imbalanced.^14^ Patients with high ratings on both variables were classified as having high prevention–adherence behaviour. Those with moderate ratings on either variable were classified as moderate, while patients with low ratings on either variable were classified as low. Determinants of composite prevention–adherence behaviour were analysed using multinomial logistic regression to identify independent predictors, with relative risk ratios (RRRs) and 95% confidence interval (CI). Although the outcome was ordinal, multinomial logistic regression was used to compare specific categories (moderate vs low and high vs low) rather than estimate a single proportional odds ratio. To minimise model instability and overfitting, the number of predictors was limited.^15^

### Ethical statement

This study was approved by the Ethics Committee of the Faculty of Medicine, Universitas Brawijaya Malang, number 55/EC/KEPK/03/2025. Respondents gave informed consent for publication prior to participating in the study.

## RESULTS

A total 147 completed both study outcomes. Some did not answer all questionnaire items, resulting in variable sample sizes across measures. The participant characteristics are as shown (https://doi.org/10.6084/m9.figshare.32928659). Just over half were male (52.38%), and the largest age group was over 45 years (39.46%). Most were married (62.59%) and served as heads of their household (41.46%). Of the 120 respondents reporting occupation, 57.50% were employed. Over half earned 1–3 million rupiahs per month (approximately 68–200 US $), had 1–2 children (57.94%), and lived with 2–3 adults (55.15%). Family functioning was predominantly good (82.76%).

Most patients had pulmonary TB (84.06%), and 29.17% reported co-morbidities. Diagnosis commonly occurred at primary health care centres or Puskesmas (62.59%). Family support during treatment was frequent (82.99%), mainly from spouses (50.43%). A majority demonstrated a positive attitude toward TB (75.34%). Most did not perceive stigma (83.04%). Over half had good TB knowledge (53.10%). Additionally, 55.86% had previously received TB-related information.

### Concordance between TB transmission-prevention behaviours and treatment adherence

The cross-tabulation showed a clear ordinal trend, where higher levels of TB transmission-prevention practices corresponded with higher treatment adherence (Table). Exact agreement rate based on raw data was 51.7%. This is consistent with 84.3% observed agreement when including near-matches the observed agreement. The observed agreement was high as most discrepancies occurred between adjacent categories (e.g., moderate vs high), indicating minor disagreement. The quadratic weighted kappa was low (κ = 0.19) due to imbalanced marginal distributions (kappa paradox). The ordinary, or unweighted, Cohen’s kappa was 0.12. In contrast, chance-corrected statistics more appropriate for ordinal imbalanced data indicated substantially higher agreement. Gwet’s AC was 0.65 (95% CI: 0.56–0.75), consistent with substantial agreement. Quadratic weighted kappa similarly reflected moderate-to-substantial agreement.

**Table.** Association between TB transmission-prevention practice and TB treatment adherence (n = 147).

TB transmission practice	TB treatment adherence			
	Low	Moderate	High	Total
	n (%)	n (%)	n (%)	n (%)
Low	1 (0.68)	4 (2.72)	3 (2.04)	8 (5.44)
Moderate	7 (4.76)	25 (27.01)	23 (15.65)	55 (37.41)
High	4 (2.72)	30 (20.42)	50 (34.01)	84 (57.14)
Total	12 (8.16)	59 (40.14)	76 (51.70)	147 (100.0)

Exact agreement = 0.51 (76/147); observed (weighted) agreement = 0.84 (95% confidence interval [CI]: 0.81–0.88); the quadratic weighted kappa was low (κ = 0.19) (95% CI: 0.03–0.35); the ordinary, or unweighted, Cohen’s kappa was 0.12; Gwet’s agreement coefficient = 0.65 (0.56–0.75).

### Determinants of a composite TB prevention–adherence behaviour

This composite measure differentiated three meaningful behavioural patterns in managing both transmission prevention and treatment adherence: 50 (34.01%) patients demonstrated high prevention–adherence behaviour, 78 (53.06%) were classified at a moderate level, and 19 (12.93%) fell into the low group (Figure). Due to the limited sample size and missing data for several predictors, only simple (unadjusted) logistic regression models were conducted. Only gender and knowledge were significantly associated with membership in the moderate versus low behaviour group (https://doi.org/10.6084/m9.figshare.32928725). Male patients were substantially less likely than females to report moderate preventive–adherence behaviour (RRR = 0.31; 95% CI: 0.10–0.93; *P* < 0.05). In contrast, patients with high knowledge levels were more likely to exhibit moderate behaviour compared with those with low-to-moderate knowledge (RRR = 3.62; 95% CI: 1.19–11.05; *P* < 0.05).

**Figure.**
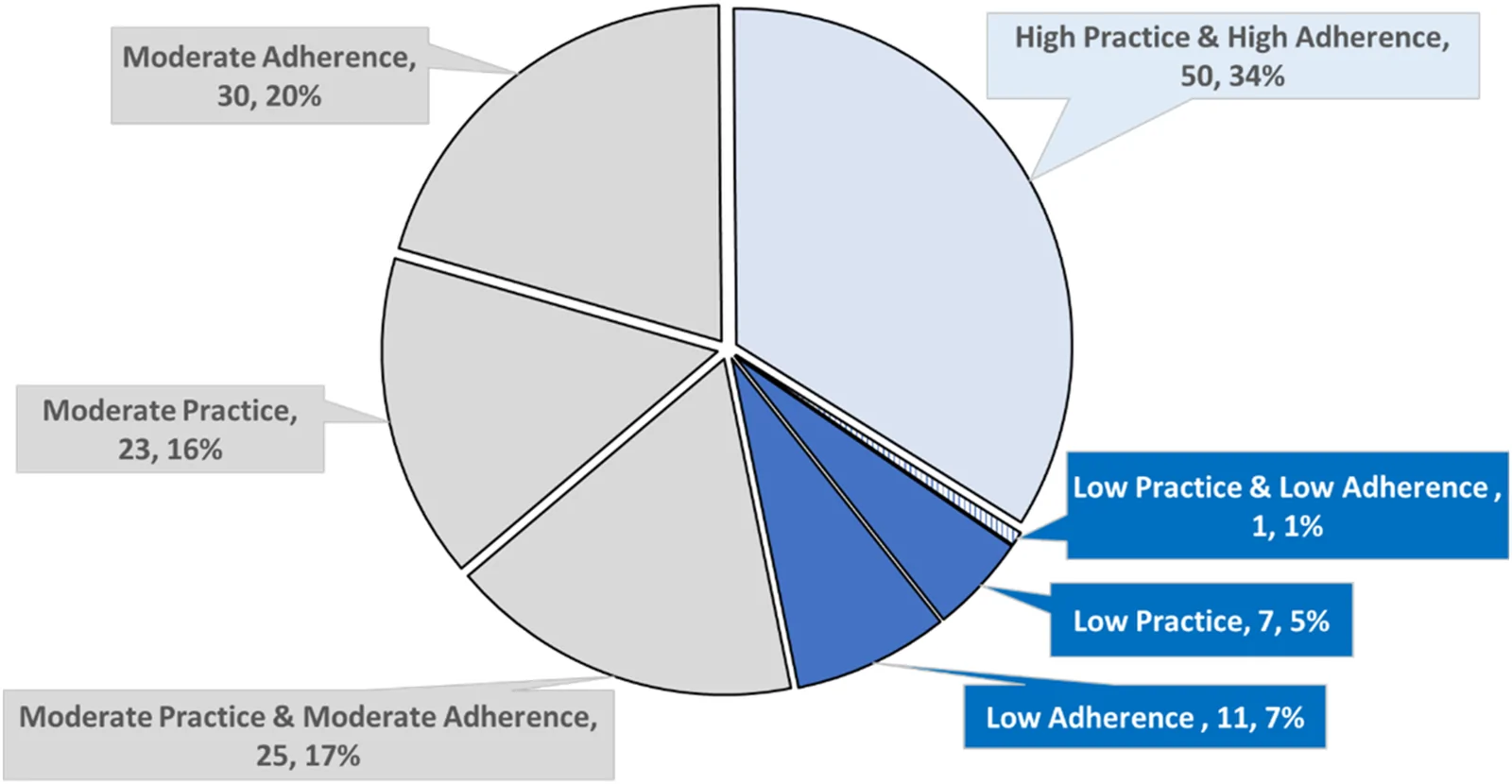
Composite TB prevention–adherence behaviour classification.

When assessing the odds of being in the high behaviour group relative to the low group, several determinants showed statistically significant associations (https://doi.org/10.6084/m9.figshare.32928725). Education, income, family function, attitude, and knowledge emerged as significant predictors. No significant associations were observed for clinical factors. Patients with 10–12 years of education were more likely to report high preventive–adherence behaviour compared with those with fewer than 10 years of education (RRR = 3.67; 95% CI: 1.01–13.34; *P* < 0.05). Likewise, patients with an annual income of 1–3 million were substantially more likely than those earning less than 1 million to report high-level behaviour (RRR = 6.00; 95% CI: 1.30–27.61; *P* < 0.05). Good family functioning was associated with higher preventive–adherence behaviour (RRR = 4.15; 95% CI: 1.09–15.83; *P* < 0.05). Patients with high attitude scores had greater odds of being in the high behaviour group compared with those with low-to-moderate attitudes (RRR = 4.47; 95% CI: 1.33–15.00; *P* < 0.05). Similarly, high knowledge levels were associated with a greater likelihood of reporting high preventive–adherence behaviour (RRR = 3.87; 95% CI: 1.21–12.40; *P* < 0.05).

## DISCUSSION

Among the 147 patients enrolled, 57.14% showed high treatment adherence and 51.70% demonstrated high transmission-prevention behaviour, indicating that about half maintained good TB-related practices. These adherence levels are comparable to findings from South Korea (56.5%),^16^ and slightly higher than China (45.7%).^17^ As emphasised by the WHO, maintaining good adherence is critical for achieving national TB programme treatment-success targets.^18^ Regarding transmission-prevention behaviour, the approximately 52% observed in this study aligns with international patterns, where the proportion reporting good or high TB prevention behaviour ranges from around 40% to >85%. A community survey in South Africa reported 55% good preventive behaviour.^19^ In Southeast Asia, Thailand reported 86.9% high prevention practice among pulmonary TB patients,^3^ while Indonesia reported moderate levels, with approximately 58% of TB patients demonstrating sufficient or good prevention behaviour.^20^ These differences largely reflect variation in study populations, measurement methods, and local implementation contexts.

This study is unique in integrating TB transmission-prevention behaviour and treatment adherence into a single composite measure, revealing moderate-to-substantial concordance between the two domains. Evidence from several countries supports the close linkage between these behaviours: in Indonesia, patients with higher adherence demonstrated significantly better prevention practices^4^; in Thailand, higher adherence and stronger health literacy aligned with higher prevention-behaviour scores.^3^ Quasi-experimental research in Indonesia also showed that behavioural-counselling interventions improved both adherence and prevention practices simultaneously,^21^ and in India, increased patient knowledge and engagement strengthened both adherence and prevention behaviour.^22^ Despite this consistent evidence, the combined analysis in the present study shows that only one third of patients achieved both high adherence and high prevention behaviour, about half were moderate, and 13% were low. This demonstrates that ‘good–good’ behaviour is far less common when the two domains are examined together, underscoring the need for integrated, patient-centred approaches to enhance both adherence and prevention practices simultaneously.

Gender was significantly associated with moderate, but not high, levels of composite TB prevention–adherence. Female patients were more likely than males to fall into the moderate category, consistent with evidence that women often engage more actively in infection-prevention practices and treatment adherence.^23^ In Indonesia, women’s caregiving roles and responsibility for household health decisions expose them to more health information and community-based education, including guidance from health workers during local gatherings.^12^ Men, in contrast, may have fewer opportunities to participate in such activities due to work-related schedules, which can contribute to treatment non-adherence.^24^ These factors likely elevate women’s adherence to a moderate level, but not necessarily to the highest level. The absence of a gender difference in the high-adherence group suggests that reaching optimal adherence depends more on stable support systems and programmatic interventions.

This study found that patient knowledge influenced both moderate and high levels of TB prevention–adherence. Over half of the participants had good knowledge, consistent with previous findings.^25,26^ Adequate knowledge strengthens patients’ understanding and health literacy about TB,^27^ which recent evidence shows is closely linked to better treatment adherence and self-management of preventive practices.^3^ Indonesian studies further support that accurate TB information improves patients’ prevention behaviours^28^ and promotes more consistent adherence. Overall, knowledge appears to play a critical role by enabling patients to recognise risks, follow treatment instructions, and implement daily prevention measures effectively.

Education was a significant factor associated with high prevention–adherence in this study. This aligns with evidence that higher educational attainment improves health literacy, understanding of healthy environments, and the ability to adopt clean and healthy living practices.^6,23^ Income also showed a significant effect, with patients in the moderate-income group more likely to report high preventive–adherence. Prior studies indicate that better financial resources reduce indirect treatment costs such as transportation, income loss, and nutritional needs which often hinder adherence,^8^ and that financial hardship is linked to non-adherence.^6,29^ In this study, only patients with 1–12 years of education (not >12 years) and those with moderate income showed significant associations with high prevention–adherence when compared with lower categories. This pattern likely reflects the small number of participants with very high education or high income, which may have limited the statistical power to detect differences in these groups.

We included family function as a predictor, as this is understudied in previous work. Family function showed a strong effect: patients from well-functioning families were nearly four times more likely to achieve high prevention–adherence than those from poorly functioning families. Supportive relationships and effective communication can enhance shared responsibility and health awareness within the household.^30^ Families also provide crucial TB information that improves patients’ understanding of symptoms, transmission, and prevention,^31^ and informational support has been linked to greater self-efficacy.^32^

Among psychosocial factors, patients with higher attitude scores were significantly more likely to achieve high prevention–adherence. Those who recognise their vulnerability to TB tend to be more motivated to carry out protective behaviours, which is consistent with earlier findings.^33^ Increased risk perception encourages patients to limit close contact and follow recommended infection-control measures.^34,35^ Perceived stigma was not significant in this study because more than 80% of participants reported no feelings of stigma. With both a generally high level of positive attitudes and low perceived stigma, patients may feel accepted and supported by their families and communities. It reduces psychological barriers and helps sustain preventive behaviours and treatment adherence.

This study had several limitations; for example, self-reported measures for both adherence and prevention behaviours introduce the possibility of recall or social desirability bias. This study demonstrated a Cronbach’s alpha coefficient of 0.66 that may reflect differences in how participants interpreted certain items or the multidimensional characteristics of the constructs being measured. Therefore, the results should be interpreted cautiously. The sample was limited to primary health care patients in Malang and may not represent patients in other health care settings or regions of Indonesia. In addition, the study did not assess health care provider–patient communication, which may influence prevention and adherence behaviours. The regression findings should be interpreted as exploratory rather than evidence of independent effects, as important confounding may exist among related variables such as education, income, knowledge, and attitude. Due to the modest sample size, missing data, and multiple predictor categories, the adjusted model may be unstable and prone to overfitting. Therefore, larger studies with more complete data are needed to confirm these associations.

## CONCLUSION

Routine interventions are needed in the form of educational programmes for patients and their families during the intensive phase of treatment, along with individualised family assistance through personalised education sessions when patients visit primary health centres to collect their medications.
